# A Matter of Trust: Building and Validating the Dental Hygienist Trust Scale

**DOI:** 10.3390/healthcare14152304

**Published:** 2026-07-31

**Authors:** Cecilia Di Cesare, Giulia Valentini, Marco Dolci, Silvia D’Agostino

**Affiliations:** Department of Medical, Oral and Biotechnological Sciences, University G. d’Annunzio, 66100 Chieti, Italy; ceciliadicesare@virgilio.it (C.D.C.); giulia.valentini@unich.it (G.V.); marco.dolci@unich.it (M.D.)

**Keywords:** patient trust, scale development, validity, dental hygienist, psychometrics

## Abstract

**Highlights:**

**What are the main findings?**

- The Dental Hygienist Trust Scale (DHTS) was successfully developed and preliminarily validated as the first instrument specifically designed to measure patient trust in dental hygienists.
- The DHTS demonstrated strong psychometric properties, including high internal consistency (Cronbach’s α = 0.88) and a unidimensional factor structure explaining 62.4% of the variance, while patients reported generally high levels of trust regardless of age or gender.

**What are the implications of the main findings?**

- The DHTS is designed to provide researchers and clinicians with a reliable instrument to assess the patient–dental hygienist relationship, addressing a significant gap in oral health literature.
- The DHTS provides researchers and clinicians with a reliable instrument to assess patients’ trust in dental hygienists, enabling standardized evaluation of the patient–dental hygienist relationship and supporting future studies investigating its impact on clinical and patient-reported outcomes.

**Abstract:**

**Introduction**: Trust is a fundamental component of the therapeutic alliance in healthcare, yet research in the dental field has traditionally focused on dentists, often neglecting the distinct role of the dental hygienist. To the best of our knowledge, no validated tools exist specifically for measuring patient trust in dental hygienists. Objectives: This study aimed to develop and validate the Dental Hygienist Trust Scale (DHTS), an instrument adapted to assess patient trust specifically toward dental hygienists. **Methods**: Following a linguistic and contextual adaptation of the original Dentist Trust Scale, a cross-sectional study was conducted at the University Dental Clinic of Chieti. Psychometric properties were assessed using Cronbach’s alpha for internal consistency and Exploratory Factor Analysis (EFA) for factorial validity. Inferential statistics included the Shapiro–Wilk test, independent samples *t*-tests, and Pearson’s correlation coefficient. **Results**: A total of 161 participants were recruited (mean age 46.7 ± 19.1 years; 52.2% female). The DHTS showed high internal consistency (alpha = 0.88) and a robust single-factor structure (62.4% of total variance). Participants reported high trust levels (mean score 3.65 ± 0.56). No significant differences were observed regarding gender (*p* = 0.218) or age (r = 0.04, *p* = 0.612). **Conclusions**: The initial psychometric evaluation of the DHTS provides a preliminarily validated instrument for assessing the patient-hygienist relationship, filling a significant gap in dental literature. Further longitudinal research is recommended to explore the temporal stability and clinical impact of these trust perceptions.

## 1. Introduction

Trust is one of the most multifaceted concepts in the social sciences. It is understood both as an essential mechanism for reducing social complexity and as a fundamental prerequisite for human cooperation in situations of vulnerability [1,2]. In psychological terms, trust is defined as a generalized expectation held by an individual that another person’s word, promise or statement is reliable [3]. In the healthcare sector, this expectation is not only an ethical prerequisite but also serves as an indispensable driver for ensuring treatment adherence and effective cooperation between patients and healthcare professionals [4]. Although numerous studies have focused on trust within primary care or public health systems, there is a growing need to explore these dynamics within highly relational specialties, such as oral health and preventive dentistry [5]. The field of dentistry occupies a distinct position within the medical landscape. According to the Ipsos Global Trustworthiness Index (2023–2024), healthcare professionals consistently top the rankings of the world’s most trusted figures, with trust scores exceeding 58%. However, regional insights highlight a complex paradox: although around 70% of the population say they are satisfied with their oral health, trust in dental professionals is no longer a static attribute, but a ‘dynamic resource’ that requires continuous validation during every clinical encounter [6,7]. In this context, the dental hygienist emerges as a key figure, acting not only as a provider of clinical care but also as a ‘health facilitator’, essential for strengthening the therapeutic alliance. Despite this crucial role, there is a lack of specific, validated tools to quantify the relationship of trust between patients and dental hygienists. Most existing instruments focus generically on doctors or the dental profession as a whole, failing to capture the unique preventive and motivational nuances of dental hygiene practice.

Defining the concept of trust remains a complex challenge: despite the extensive literature, significant conceptual fragmentation persists in the academic debate due to the variety of interdisciplinary approaches adopted. This uncertainty often stems from a lack of integration between different schools of thought and from the fact that many definitions have been shaped more by specific empirical research needs than by a unified theoretical framework [8]. This lack of clarity has also influenced health research, where the focus has been predominantly on measuring levels of trust rather than on understanding the deeper nature of trust relationships [9]. To analyze the complex dynamics governing the relationship with the healthcare system, it is therefore necessary to return to the fundamentals of the concept. Among the various perspectives available, a particularly effective definition is that proposed by the Organisation for Economic Co-operation and Development (OECD), which describes trust as: ‘a person’s belief that another person or institution will act in a manner consistent with expectations of positive behavior’ [10,11]. This formulation has the merit of capturing both behavioral and attitudinal aspects, providing a clear and intuitive basis for breaking down the general notion of trust into more specific analytical categories. However, in the clinical context, it is essential to recognize that the object of trust is not a static entity; on the contrary, it varies significantly depending on the specific type of healthcare professional involved and the unique nature of their professional practice [12,13]. Regardless of the specific theoretical framework, scholars agree that trust inherently involves uncertainty and vulnerability.

Deciding to trust implies a willingness to make oneself vulnerable to the actions of another party, regardless of one’s ability to control them [10]. Since the person placing trust cannot predict with certainty whether the recipient will honor that trust, they are exposed to both material and emotional risks [14,15]. According to Luhmann, the prerequisite for trust is familiarity: detailed information acquired from past interactions that helps reduce social complexity [1]. However, trust is not merely a retrospective inference; it is a forward-looking ‘act of faith’ that shapes the future by acting as if certain negative possibilities will not occur [16,17]. This process is not purely cognitive; it encompasses the emotional and psychological spheres, which are fundamental in healthcare [18]. In this context, the distress of illness can trigger trust as a coping mechanism or, conversely, negative emotions can diminish perceived reliability regardless of objective evidence [19]. Finally, trust has a temporal and processual dimension; it is subject to development and learning [20]. Rather than a static stage, it should be viewed as ‘trusting’—a process through which individuals generate, maintain or lose their willingness to show vulnerability [17]. Despite growing interest in trust in general medicine, research specifically focused on the dental context remains relatively underdeveloped. Historically, studies in this field have relied on single-item questions or adapted versions of the General Scale of Trust in Physicians, often without taking into account the unique nature of the dental encounter [12].

Early research has shown that trust is a primary determinant of dental attendance, with lower levels of trust being significantly associated with high dental anxiety and avoidance behaviors [21]. Furthermore, researchers have highlighted that trust in dentistry is uniquely linked to fear of pain and the perception of transparency regarding treatment costs—factors that distinguish it from trust in general medicine [22]. Recent literature further characterizes trust as a dynamic and adaptive process involving a ‘leap of faith’ on the part of the patient, in which each clinical encounter serves as ongoing ‘proof’ of the practitioner’s reliability [23,24]. Emerging evidence suggests that the communication of trust and the perceived quality of service account for a significant part of patient satisfaction, with patients increasingly valuing digital transparency and technological competence as indicators of clinical reliability [25]. Recent comprehensive reviews highlight that there is still no consensus on a preferred assessment tool for dental trust, pointing to a significant gap in in-depth investigations [26]. Although trust is now recognized as a multidimensional concept, its empirical measurement remains limited. For example, the Dentist Trust Scale—one of the most widely used tools for assessing dentists’ honesty and competence—continues to focus exclusively on the doctor-patient relationship [24]. This creates a significant gap in the literature: the role of the dental hygienist, whose practice focuses on long-term prevention, motivational interviewing and repeated behavioral interventions, remains virtually unmeasured.

This lack of data appears to be attributable to several factors. Firstly, there is an almost total absence of measurement tools specifically designed for this profession; indeed, most existing studies adapt scales originally designed for general medicine. Furthermore, as highlighted by recent reviews, there is still no scientific consensus on standardized tools capable of capturing the nuances of the relationship with technical healthcare professions [27]. Current models overlook the paradox of prevention and the specific nature of the dental hygienist’s role. Whilst medical trust is usually studied in acute care settings, the practice of oral hygiene is based on a long-term therapeutic alliance and on the shift from mere compliance to active patient involvement (concordance) [27]. Therefore, remains a need for specific self-assessment scales and measures of the perception of the hygienist’s communicative and educational skills, distinct from those of the rest of the dental team [28]. Finally, the literature has rarely quantified the impact of conflicting signals perceived by the patient within the dental practice. Trust in the hygienist can, in fact, be undermined or redefined by discrepancies in information between different professionals [29], making trust in this specific role an independent variable that merits a dedicated and culturally validated investigation. Despite the growing recognition of trust as a key determinant of patient-centered care, no validated instrument is currently available to specifically assess patients’ trust in dental hygienists. This represents an important gap in both clinical practice and oral health research, as the preventive and educational role of dental hygienists differs substantially from that of dentists and requires dedicated evaluation. Therefore, the primary objective of this study was to develop and perform an initial psychometric evaluation of the Dental Hygienist Trust Scale (DHTS), providing the first standardized instrument specifically designed to assess patients’ trust in dental hygienists for use in clinical practice and future research. Accordingly, we sought to examine whether the DHTS demonstrated acceptable internal consistency and an interpretable factor structure in a sample of adult dental patients.

## 2. Materials and Methods

### 2.1. Study Design and Instrument Development

Prior to the commencement of the study, the questionnaire underwent a pilot-testing phase to assess clarity, readability, and ease of navigation. This validation panel consisted of one dentist, one dental hygienist, one maxillofacial surgeon, and four final-year dental hygiene students. Feedback from this panel was used to refine the item phrasing, ensuring that the terminology was accessible and relevant to the dental hygiene practice context. The questionnaire was finalized with an estimated completion time of approximately two minutes. Regarding the study design, a convenience sampling method was employed. To ensure sufficient statistical power for the psychometric validation—specifically to conduct a reliable Exploratory Factor Analysis (EFA)—a minimum sample size of 150 participants was targeted, based on the recommendation of a 10:1 ratio of subjects to items.

### 2.2. Participants and Data Collection

The survey was administered to patients attending the University Dental Clinic at the University “G. d’Annunzio” of Chieti. Inclusion criteria were defined as follows: individuals aged 18 or older who were proficient in the Italian language. No specific exclusion criteria were applied beyond failure to meet the inclusion criteria. Individuals younger than 18 years of age, those unable to understand the Italian language, and those who declined to provide informed consent were not eligible to participate. In addition to the items of the DHTS, demographic data including age and gender were collected for each participant. All subjects provided informed consent in accordance with the Declaration of Helsinki. Data collection was conducted via an anonymous Google Form, presented on a tablet, while patients were in the waiting area prior to their scheduled dental hygiene appointment. To mitigate social desirability bias—the tendency of respondents to provide answers perceived as socially acceptable—a standardized explanation of the survey’s structure and purpose was provided. Following this briefing, participants were left unobserved to ensure privacy and to facilitate independent completion. However, a member of the research team remained available in the vicinity to provide technical assistance, ensuring that no influence was exerted on the participants’ responses.

### 2.3. Data Management

All data were collected anonymously via the secure Google Form platform. To ensure complete participant anonymity, the survey was configured to collect responses without recording any personally identifiable metadata, such as email addresses or unique device identifiers. The resulting dataset was managed within the secure environment of the University’s Google Workspace, ensuring restricted access to the primary research team. Data were exported directly from the platform into a structured format for statistical analysis, preventing any manual data entry and thereby minimizing the risk of transcription errors. In accordance with the General Data Protection Regulation (GDPR) and institutional guidelines, the digital dataset will be stored securely for a period of five years before being permanently deleted.

### 2.4. Ethical Aspect

The study protocol received formal approval from the Departmental Review Board (Approval No. 14/2026). All participants were fully informed that their responses would be handled with strict confidentiality and that they reserved the right to withdraw from the research at any stage without prejudice. It was explicitly stated that individual anonymity would be maintained in all reported findings. Written informed consent was obtained from all participants prior to their involvement. No financial compensation or other incentives were provided for participating in this study.

### 2.5. Scale Items and Description

To assess patient trust in dental hygienists, the research team adapted the Dental Trust Scale (DTS), originally developed by Armfield et al. in 2017 [21]. This process resulted in an 11-item Dental Hygienist Trust Scale (DHTS). Evidence for the original scale’s psychometric robustness includes a single-factor structure and high internal reliability (α = 0.92). Additionally, the scale demonstrated predictive validity; higher trust scores were correlated with greater compliance with treatment recommendations, fewer interpersonal conflicts, and increased continuity of care. The DTS was selected because it is one of the few validated instruments specifically designed to assess trust in dental professionals and has demonstrated excellent psychometric properties in previous research. The adaptation process primarily involved substituting the term ‘dentist’ with ‘dental hygienist,’ accompanied by minor lexical adjustments to ensure terminological consistency within the new clinical context. Mirroring the structure of the original scale, the adapted items assess multiple facets of interpersonal trust—specifically fidelity, conflicts of interest, professional competence, and honesty—alongside global trust. This multidimensional approach is grounded in an extensive review of existing literature [30]. The specific item phrasing for the DHTS is presented in Table 1, The response format consisted of a five-point Likert scale (1 = ‘Strongly disagree’, 5 = ‘Strongly agree’), designed to capture the intensity of the respondents’ agreement. Items 2 and 7 were negatively worded and were reverse-coded prior to statistical analyses so that higher scores consistently reflected higher levels of trust.

### 2.6. Statistical Analysis

Descriptive statistics were employed to summarize participant demographics and to provide an overview of item distributions and mean scores. Descriptive statistics were employed to summarize participant demographics and to provide an overview of item distributions and mean scores. The overall DHTS score was calculated as the mean of the 11 item scores for each participant, and descriptive statistics were subsequently computed on these participant-level composite scores. The internal consistency of the DHTS was assessed using Cronbach’s alpha coefficient to evaluate the consistency of responses across the scale items; a value ≥ 0.70 was considered the threshold for acceptable reliability. To examine the underlying dimensionality of the DHTS and verify whether the items measured a common latent construct, an Exploratory Factor Analysis (EFA) was performed using principal axis factoring. To explore the underlying dimensionality of the DHTS—as previously outlined in the study design—an EFA was performed using principal axis factoring. The number of factors to be retained was determined by the eigenvalue > 1 criterion, supported by visual inspection of the scree plot, to ensure a robust and parsimonious factor structure. Finally, to investigate the influence of demographic characteristics on the level of trust, we examined the associations between DHTS mean scores and participant variables. Independent samples *t*-tests were used to compare mean scores between genders, while the relationship between age and trust scores was evaluated using Pearson’s correlation coefficient. Data processing was performed using spreadsheet software. Descriptive and inferential statistical analyses were conducted using GraphPad Software (Version 11.0.2, San Diego, CA, USA), whereas the EFA was conducted using the jamovi statistical platform (Version 2.3, The jamovi project, 2024).

## 3. Results

### 3.1. Descriptive Analyses

A total of 161 participants were recruited for this study. Regarding the demographic characteristics of the sample, the mean age of the respondents was 46.7 years (SD = 19.1), with an age range spanning from 18 to 86 years. In relation to the gender distribution, 52.2% participants (84/161) were female, while 47.8% participants (77/161) were male. The descriptive statistics for each of the 11 items of the DHTS, including mean scores, standard deviations, and response frequencies across the five-point Likert scale, are presented in Table 2.

Mean scores ranged from 3.41 (Items 2 and 6) to 3.91 (Item 8), reflecting a generally positive orientation toward trust. Overall, the frequency distribution indicates that participants leaned toward the higher end of the scale, as evidenced by the high concentration of ratings in categories 4 and 5 for the majority of the items. It is worth noting that some items, particularly Item 6 (35.9% neutral) and Item 7 (32.5% neutral), elicited a non-negligible proportion of neutral responses, which may warrant further investigation regarding patient ambiguity on these specific aspects of the relationship. The generally high DHTS scores may also suggest the presence of a ceiling effect, potentially limiting the instrument’s ability to discriminate among individuals with very high levels of trust. Future studies involving more heterogeneous populations should further investigate this aspect. The overall mean score for the DHTS was 3.65 (SD = 0.56), indicating a high and consistent level of trust perceived by the participants toward the dental hygienist. Notably, no participants reported extreme dissatisfaction (scores below 2.0), suggesting a generally positive trend in the population examined. When analyzing the data by gender, female participants reported a mean score of 3.72 (SD = 0.55), while male participants showed a mean score of 3.61 (SD = 0.58). Despite these slight variations, both groups demonstrated a consistently high level of trust, with the vast majority of responses falling in the positive range of the scale. Overall, the findings indicate no significant gender-based disparity in trust levels.

### 3.2. Inferential Statistics

**Figure 1:**
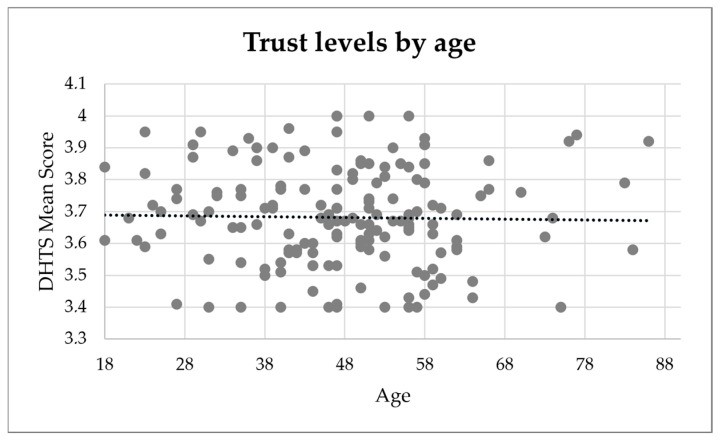
Scatter plot of the relationship between participants’ age and DHTS scores. The data points show a dispersed distribution, indicating no significant linear correlation between age and perceived trust.

Prior to performing inferential analyses, the normality of the data distribution was assessed using the Shapiro–Wilk test, which confirmed that the data were normally distributed (*p* > 0.05). Subsequently, an independent samples Student’s *t*-test was conducted to compare scores by gender. The results indicated that the difference between groups was not statistically significant (t(159) = 1.24; *p* = 0.218) with a small effect size (Cohen’s d = 0.12, 95% CI [−0.15, 0.39], suggesting that gender does not significantly influence the levels of trust toward the dental hygienist in this sample. Finally, a Pearson correlation analysis was employed to examine the relationship between age and trust levels, revealing no significant linear correlation (r = 0.04, *p* = 0.612, 95% CI [−0.11, 0.19]). This absence of a linear relationship is visually confirmed by the scatter plot shown in Figure 1. Overall, these findings suggest that the perceived trust toward the dental hygienist is consistent across different demographic groups.

### 3.3. Reliability and Factorial Validity

**Figure 2:**
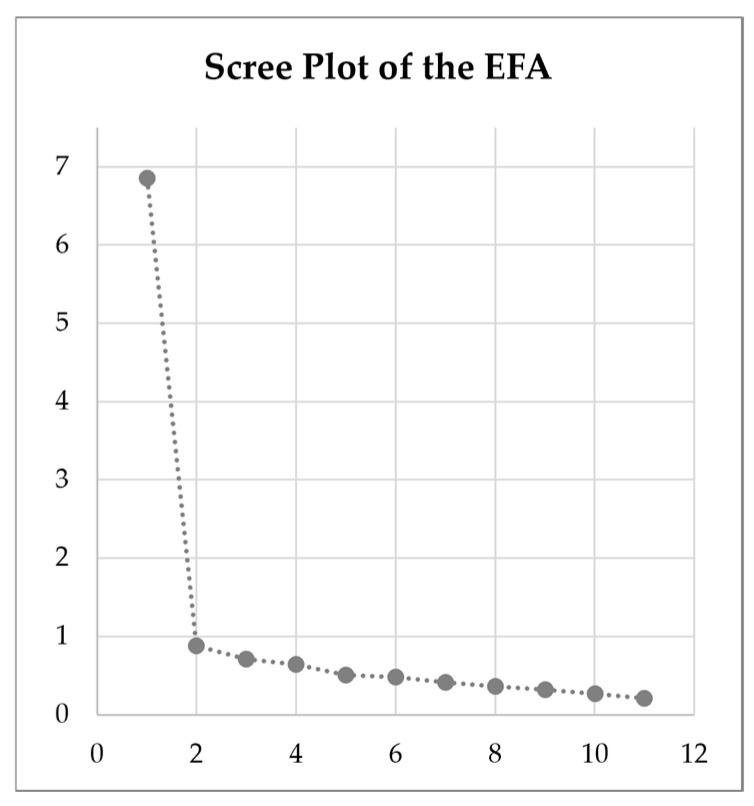
Scree plot of the EFA supporting the single-factor structure of the DHTS. The distinct ‘elbow’ after the first factor, along with the high initial Eigenvalue (6.85) and the subsequent drop below 1.0, confirms the unidimensional nature of the scale.

To evaluate the psychometric properties of the DHTS, the internal consistency was assessed using Cronbach’s alpha coefficient. The analysis yielded a value of 0.88, indicating high reliability and strong internal consistency of the scale items. Given the cross-sectional nature of the study, test–retest reliability was not assessed, focusing the validation on the internal coherence of the items. Subsequently, to investigate the underlying dimensionality of the scale, an EFA was conducted using principal axis factoring. The analysis confirmed a single-factor structure, as all 11 items exhibited significant factor loadings on a single component (eigenvalues > 1). This single factor accounted for 62.4% of the total variance, confirming the unidimensional nature of the DHTS in measuring patient trust toward dental hygienists. The unidimensionality of the scale was further supported by the inspection of the scree plot (Figure 2), which showed a distinct ‘elbow’ after the first factor, confirming that a single-factor solution is the most appropriate representation of the data. Detailed psychometric results are provided in Table A1.

## 4. Discussion

### 4.1. Discussion of Findings

The primary objective of this study was to develop and psychometrically validate the DHTS, thereby addressing a prominent gap in oral health literature regarding the quantitative assessment of trust in technical and preventive healthcare figures. Our findings demonstrate that the DHTS is a highly reliable, unidimensional instrument possessing strong internal consistency (α = 0.88) and a robust single-factor structure that accounts for 62.4% of the total variance. These psychometric properties closely replicate the structural behavior of the original Dentist Trust Scale (DTS) developed by Armfield et al. [21] (α = 0.92), as well as earlier foundational measures of physician trust, confirming that the lexical and contextual adaptation maintained the structural integrity of the baseline instrument while successfully pivoting to the dental hygiene framework.

A compelling finding of this study is the confirmation of a single-factor structure for the DHTS. The original DTS was constructed around distinct theoretical dimensions of interpersonal trust, specifically fidelity, professional competence, honesty and global trust. The fact that all 11 items loaded onto a single component confirms that, in the patient’s perception, these dimensions are not statistically independent constructs. In line with the psychometric literature on medical trust, this unidimensionality suggests that patients do not distinctly separate a practitioner’s technical competence from their honesty or fidelity during a clinical encounter [12]. Two distinct hypotheses can explain this phenomenon. From an aggregate perspective, it could be argued that patients lack the technical criteria to conceptually isolate these traits. Alternatively, and more plausibly, a patient’s global trust acts as an overarching psychological lens. This global perspective strongly colors and influences individual behavioral dimensions to the point where they can no longer be evaluated apart from a macro-evaluation of the professional. For the dental hygienist, whose clinical scope relies heavily on repeated preventive appointments, this overarching trust mechanism acts as a critical buffer during care delivery.

Descriptive analyses revealed a generally positive orientation toward dental hygienists, yielding an overall mean DHTS score 3.65 + 0.56. The highest scores were observed in Item 8 (Mean = 4.0) and Item 3 (Mean = 3.9). These elevated scores reflect the unique clinical role of the dental hygienist, whose professional practice is inherently anchored in long-term prevention, repetitive behavioral interventions, and detailed patient education. In health literature, such high ratings of thoroughness and dedication are strongly tied to the perception of service quality and clinical competence, which function as critical determinants of overall patient satisfaction [31].

However, a more nuanced pattern emerged at the lower end of the spectrum. Item 10 generated the lowest mean score (3.4) and the widest response variability. This finding perfectly aligns with international dental literature suggesting that absolute trust is rarely a static or unconditional attribute [32]. Patients may harbor slight reservations regarding total absolute transparency, a phenomenon frequently connected in dental research to underlying concerns regarding the clarity of treatment costs, financial conflicts of interest, or digital transparency.

Furthermore, significant neutral or midpoint responses were observed for Item 6 (35.9%) and Item 7 (32.5%). This trend closely mirrors findings from the development of the original DTS, where approximately one-quarter to one-third of participants consistently selected neutral midpoints. The relatively higher proportion of neutral responses observed for the negatively worded item may reflect, at least in part, the greater cognitive complexity typically associated with reverse-worded statements. Future studies should investigate whether alternative positively worded formulations improve the psychometric performance of these items. In psychometric terms, a high frequency of neutral responses may signal a degree of consumer ambiguity regarding specific professional behaviors [12]. It also highlights a critical segment of the population that has not developed explicit distrust, but instead maintains a passive, uncommitted stance.

The item distribution underscores a fundamental truth within clinical dental research: interpersonal communication and rapport are more critical in establishing trust than the objective technical outcomes of treatment. This emphasizes the need for dental hygienists to enhance patient-centered communication competencies to fully bridge the gap between compliance and active concordance.

In historical evaluations of the DTS, lower trust scores were not strongly linked to clinical triggers like a past history of dental pain. Instead, they were heavily associated with negative interpersonal experiences, such as having previously felt embarrassed or having experienced personal friction with the practitioner [33]. This reality carries profound implications for the dental hygiene profession. Because the hygienist’s clinical framework is rooted in shifting patients from mere compliance to active concordance, the quality of the communicative exchange dictates the therapeutic alliance. Establishing a strong rapport does not merely improve patient satisfaction; it actively modifies the patient’s psychological management. Staff and practitioner behaviors serve as primary tools for provoking or ameliorating dental anxiety. Building a trusting relationship provides the actual foundation for reducing avoidance behaviors and mitigating fear [34].

Inferential statistics demonstrated that trust perceptions remained remarkably uniform across demographic cohorts. The Shapiro–Wilk test confirmed the normality of the data, and the subsequent Student’s *t*-test revealed no significant difference in trust scores between female (3.72 + 0.55) and male (3.61 + 0.58) participants (*p* = 0.348). Similarly, Pearson correlation analysis indicated an absolute absence of a linear relationship between age and trust levels (r = 0.04, *p* = 0.612). The lack of significant association between demographic variables and trust levels suggests that, in this sample, the therapeutic alliance fostered by dental hygienists does not significantly vary across age and gender.

While in other medical contexts age or gender may influence trust dynamics, in this specific sample, no such associations were observed. These findings suggest that the routine and preventative nature of dental hygiene maintenance visits may provide a consistent basis for building rapport, though this observation is limited to the demographic characteristics of the participants included in this study.

Finally, the cross-sectional data from the DHTS must be interpreted in light of broader institutional and systemic dynamics. Literature shows a powerful correlation between a patient’s trust in a specific, individual practitioner and their trust in the health profession as a whole [35]. This structural link points to inevitable “flow-over effects”. A single negative or alienating experience with an individual dental professional can easily trigger a macro-level erosion of trust toward the entire dental team, inducing protective behaviors such as “practitioner shopping” or the complete abandonment of a regular dental home.

Conversely, positive reinforcement during regular, non-acute dental hygiene visits can act as an entryway to rebuilding trust in the wider oral healthcare system [36]. Because a lack of trust significantly increases the risk of delayed dental visiting, treatment non-adherence, and interpersonal disputes, utilizing the DHTS as an early screening tool provides a clear pathway toward patient-centered care and improved public health outcomes [37]. Ultimately, the validation of the DHTS underscores that trust in the dental hygienist operates as an independent, quantifiable variable within the dental team. Because conflicting signals or information discrepancies between different professionals within the same dental practice can easily undermine patient confidence, having a specific self-assessment metric allows clinics to actively monitor the psychosocial foundation of their preventive care delivery.

Fostering high trust through this preliminarily validated instrument may potentially contribute to mitigating dental anxiety, reducing avoidance behaviors, and facilitating long-term patient adherence to oral health regimens; these relationships remain to be investigated in future clinical studies.

From the perspective of positive psychology, trust can be regarded as a key relational resource that promotes constructive interactions between healthcare professionals and patients. Within dental hygiene practice, fostering trust may contribute to strengthening the therapeutic alliance, encouraging patient engagement, and supporting a more positive healthcare experience. Although the present study focused on the initial psychometric evaluation of the DHTS rather than on clinical outcomes, the availability of a standardized instrument to assess trust provides an important foundation for future research exploring positive psychological constructs in preventive oral healthcare.

### 4.2. Limitations

Several limitations should be acknowledged when interpreting the findings of this study. First, the study design was cross-sectional, which precludes the establishment of causal relationships between variables; therefore, longitudinal research is needed to observe how trust levels evolve over time. Second, test–retest reliability was not assessed because of the cross-sectional design of the study. Consequently, the temporal stability of the DHTS remains to be established. Future longitudinal studies should evaluate the reproducibility of the instrument over time before its performance in repeated assessments can be fully characterized. However, the high internal consistency observed (alpha = 0.88) supports the scale’s reliability in a single-measurement context. Furthermore, convergent and discriminant validity were not assessed because no additional validated instruments measuring theoretically related or unrelated constructs were administered. Future studies should address these psychometric properties to provide a more comprehensive validation of the DHTS. Finally, although the questionnaire was completed anonymously and in the absence of direct observation, administration immediately before the scheduled dental hygiene appointment may have influenced participants’ responses through social desirability or courtesy bias. Consequently, this potential source of bias cannot be excluded. Future studies should investigate whether the psychometric performance of the DHTS remains consistent across different modes and timings of administration, including post-appointment and remote settings.

In addition, participants were recruited using convenience sampling from a single university dental clinic. Consequently, the findings may not be fully generalizable to other clinical settings or populations. Future multicenter studies involving more heterogeneous samples are warranted to further evaluate the external validity of the DHTS. 

The present study provides preliminary evidence supporting the internal structure of the DHTS. However, additional aspects of construct validity, including convergent and discriminant validity, remain to be established through future validation studies employing external comparison measures. Moreover, the present study did not include variables such as dental anxiety, previous dental experiences, or other factors theoretically associated with patient trust. Consequently, the ability of the DHTS to discriminate between groups expected to differ in trust could not be evaluated. Future studies should investigate this aspect to provide additional evidence of construct validity. Although the adapted questionnaire underwent expert review and pilot testing, a formal cognitive debriefing process was not conducted. Future validation studies should include cognitive interviewing techniques to further evaluate the semantic interpretation and conceptual equivalence of the questionnaire items.

### 4.3. Clinical Relevance

The availability of a preliminarily validated instrument to measure trust specifically toward dental hygienists carries significant clinical implications. The present study provides the first preliminary validated instrument specifically designed to assess patients’ trust in dental hygienists. The demonstrated internal consistency and unidimensional structure of the DHTS support its use as a standardized measure of the patient–dental hygienist relationship in both clinical practice and research. The availability of such an instrument may facilitate the evaluation of communication strategies and patient-centered interventions aimed at strengthening the therapeutic relationship. Future studies should investigate whether DHTS scores are associated with clinically relevant outcomes, including treatment adherence, dental anxiety, patient satisfaction, and preventive oral health behaviors.

It should also be acknowledged that the professional role, responsibilities, and degree of autonomy of dental hygienists vary across countries according to national regulations and healthcare systems. Consequently, although the DHTS demonstrated satisfactory preliminary psychometric properties in the present Italian sample, its application in other cultural and professional contexts should be preceded by appropriate cross-cultural adaptation and validation studies to ensure semantic, conceptual, and contextual equivalence.

## 5. Conclusions

The initial psychometric evaluation of the DHTS provides an instrument to evaluate the patient-hygienist relationship. Our results reveal generally high levels of trust, consistent across different age groups. To the best of our knowledge, this is the first study to validate a trust scale specifically tailored for dental hygienists, filling a significant gap in current dental literature. While these findings establish a positive baseline, further longitudinal research is needed to explore the factors influencing patient trust and its long-term impact on oral health behaviors.

## Figures and Tables

**Table 1:** Items of the DHTS. The table lists the 11 items developed to assess patient trust within the clinical setting.

Item	
1	Dental hygienists care about their patients’ health just as much or more as their patients do.
2	Sometimes dental hygienists care more about what is best for them than about patients’ dental needs.
3	Dental hygienists are extremely thorough and careful.
4	You completely trust dental hygienists’ decisions about which dental treatments are best.
5	Dental hygienists are totally honest in telling their patients about all the different treatment options available for their conditions.
6	Dental hygienists think only about what is best for their patients.
7	Sometimes dental hygienists do not pay full attention to what patients are trying to tell them.
8	Dental hygienists always use their very best skill and effort on behalf of their patients.
9	You have no worries about putting your oral health in the hands of the dental hygienist.
10	A dental hygienist would never mislead you about anything.
11	All in all, you trust dental hygienists completely.

**Table 2:** Item-level descriptive statistics and response frequencies for the DHTS. Response frequencies for Items 2 and 7 are presented after reverse coding.

Item	Mean	SD	Response Frequencies (%)				
			1	2	3	4	5
1	3.84	0.95	3.1	4.5	27.8	36.7	27.9
2	3.41	1.22	6.2	18.0	26.0	27.8	22.0
3	3.83	0.86	1.1	6.1	29.9	40.0	22.9
4	3.64	0.95	3.1	9.5	28.9	36.8	21.7
5	3.48	1.11	3.8	12.7	34.2	29.5	19.8
6	3.41	1.01	3.1	14.5	35.9	31.4	15.1
7	3.39	1.15	6.4	14.6	32.5	26.7	19.8
8	3.91	0.84	1.0	3.6	23.9	46.1	25.4
9	3.88	1.06	3.0	7.4	18.8	40.1	30.7
10	3.84	1.06	4.6	7.4	19.1	37.6	31.3
11	3.52	1.01	3.4	12.6	28.8	39.0	16.2

## Data Availability

The data presented in this study are available on request from the corresponding author due to privacy reasons.

## Abbreviations

The following abbreviations are used in this manuscript:

OECD	Organisation for Economic Co-operation and Development
EFA	Exploratory Factor Analysis
DHTS	Dental Hygienist Trust Scale
GDPR	General Data Protection Regulation
DTS	Dentist Trust Scale

## Appendix A

### Appendix A.1. Psychometric Details

The following table presents the psychometric results for the 11 items of the DHTS, derived from the Principal Axis Factoring analysis. The extraction was supported by an overall KMO measure of 0.88 and a significant Bartlett’s test of sphericity (χ^2^(55) = 845.2, *p* < 0.001). A single-factor structure was retained, explaining 62.3% of the total variance, with an eigenvalue of 6.85 for the extracted factor.

Detailed psychometric results, including individual factor loadings and communalities are provided in Table A1.

**Table A1:** Item-level factor loadings and communalities for the single-factor solution of the DHTS.

Item	Factor Loading	Communality (h^2^)
1	0.81	0.65
2	0.76	0.58
3	0.83	0.69
4	0.73	0.53
5	0.86	0.74
6	0.76	0.62
7	0.71	0.50
8	0.82	0.67
9	0.77	0.59
10	0.80	0.64
11	0.75	0.56

### Appendix A.2. Analytical Workflow

Data were analyzed using jamovi (v. 2.3.21) via the Principal Axis Factoring extraction method, with no rotation applied. Factor retention was based on the Kaiser criterion (eigenvalues > 1) and scree plot inspection. Missing data were handled via listwise deletion.
